# Children who develop celiac disease are predicted to exhibit distinct metabolic pathways among their gut microbiota years before diagnosis

**DOI:** 10.1128/spectrum.01468-24

**Published:** 2025-02-04

**Authors:** Kristina Kelley, Dogus Dogru, Qian Huang, Yi Yang, Noah W. Palm, Emrah Altindis, Johnny Ludvigsson

**Affiliations:** 1 Boston College Biology Department, Chestnut Hill, Massachusetts, USA; 2 Department of Immunobiology, Yale University School of Medicine, New Haven, Connecticut, USA; 3 Crown Princess Victoria’s Children’s Hospital, Region Östergötland, Linköping, Sweden; 4 Division of Pediatrics, Department of Biomedical and Clinical Sciences, Linköping University, Linköping, Sweden; China Agricultural University, Beijing, China

**Keywords:** microbiome, celiac disease, bile acid, taurodeoxycholic acid, metabolites, IgA sequencing, gut microbiota, metabolomics

## Abstract

Celiac disease (CD) is an autoimmune disease caused by a loss of gluten tolerance in genetically predisposed individuals. While 30%–40% of people possess the predisposing alleles, only 1%–2% are diagnosed with CD, suggesting that environmental factors are involved in disease pathogenesis. To determine an association between pediatric CD and the gut microbiome, we analyzed fecal samples from a prospective cohort study (ABIS). These samples were collected from children who later developed CD (CD progressors) and age-matched healthy children (at ages 1, 2.5, and 5) with similar HLA genotypes, breastfeeding durations, and gluten exposure times. We previously reported gut microbiome differences at ages 2.5 and 5 in this cohort; here, we present findings from samples collected at age 1 (*n* = 5). We identified 14 ASVs differing significantly between CD progressors and controls, including taxa linked to CD pathogenesis. CD progressors had increased Firmicutes and higher alpha diversity in IgA− bacteria. Using PICRUSt, we analyzed metabolic pathways enriched in CD progressors compared to controls at ages 1, 2.5, and 5 (*n* = 5–16), revealing enriched inflammatory and pathogenic pathways potentially contributing to CD-related immune dysregulation. While results are based on the primary EdgeR analysis, we also applied a non-parametric method of statistical analysis, reporting those results with supplementary figures. In conclusion, our findings suggest distinct metabolic pathways enriched in the gut microbiome of CD progressors years before diagnosis, which could inform targeted therapeutics for CD. As discussed in the limitations section, this small pilot study should be replicated with larger sample sizes for broader generalization.

**IMPORTANCE:**

We analyzed gut microbiome data from children who later developed celiac disease (CD progressors) compared to healthy children in the first 5 years of life. Using fecal samples corresponding to the three phases of gut microbiome development, we uncovered enriched functional microbial pathways in CD progressors at age 1. Some of these pathways, implicated in bacterial pathogenesis, microbiota modulation, and inflammation, have been correlated with CD. We also identified taxa in CD progressors at age 1 including *Lachnospiraceae*, *Alistipes*, and *Bifidobacterium dentium* that were previously associated with CD. These findings suggest a potential role for these taxa and enriched pathways in pediatric CD onset years before diagnosis, highlighting potential for early interventions. While the findings of this exploratory study should be validated with larger sample sizes, our study suggests microbial metabolic pathways related to CD onset, enhancing our understanding of CD pathogenesis and the role of gut microbiome-mediated early alterations.

## INTRODUCTION

Celiac disease (CD) is an autoimmune disease triggered by the ingestion of gluten in genetically predisposed individuals. It is characterized by small intestinal inflammation that leads to villous atrophy. However, the mechanism(s) leading to disease development are not understood. CD affects 1%–2% of the population worldwide, making it one of the most common lifelong disorders in people of all ages, and its prevalence and incidence continue to increase (1–3). In addition to adult cases, there is a rising incidence of pediatric CD in the United States and Europe (4, 5). In a local example in the United States (Denver, Colorado), the prevalence of CD by age 15 was 3.1%, which is twofold higher than the estimation in the adult population (3).

Twenty to forty percent of the population possess human leukocyte antigen (HLA)-DQ2 or (HLA)-DQ8 haplotypes that bind and present gliadin, the gluten component acting as an antigen in CD. However, only 1%–2% of individuals develop CD (6). Previous research including discordant results for CD in identical twin studies (7, 8), along with immigrant studies (9) support the indication that environmental factors play a significant role in CD pathogenesis (9). Currently, the only way to manage CD is strict adherence to a gluten-free diet (GFD), but 20%–40% of patients do not respond to the GFD and continue to have persistent or recurrent symptoms (10–12). Additionally, a GFD is difficult to adhere to and can negatively affect child development and quality of life (12–15). Therefore, there is a need to define environmental factors that play a role in CD onset and to understand the molecular mechanisms underlying CD etiology.

The gut microbiome is directly involved in the development and maintenance of the immune system and host immune responses (15). Furthermore, gut microbiota is associated with autoimmune diseases via direct and indirect interactions with innate and adaptive immune cells (16, 17). This interaction potentially results in loss of immune tolerance, chronic inflammation, and immune response against host tissues (18, 19). We and others previously reported altered microbial (20–27) and metabolite composition (22, 27–31) in both infant and adult CD patients as well as in those at risk for developing the disease. Despite significant differences in the results of these studies on the patients, one common conclusion is that CD patients have an altered gut microbiome compared to healthy controls. This includes a decrease in some beneficial genus or species at the ASV level known to have anti-inflammatory properties, such as *Bifidobacteria* (24, 26). Some studies have also reported an increase in some bacterial species known to contribute to intestinal permeability, such as *Bacteroides* species (21, 24, 32, 33). These findings suggest that gut microbiota may play an important role in CD pathogenesis; however, no causal link has been identified yet.

In our recent study (34), we investigated the role of gut microbiota and microbial metabolites in CD onset by analyzing fecal and plasma samples from CD progressors and controls in the All Babies in Southeast Sweden (ABIS) cohort. We observed that CD progressors have a distinct gut microbiota composition at ages 2.5 and 5, years before diagnosis. We performed IgA sequencing and discovered that not only did CD progressors have more bacteria coated in IgA, but the bacteria were also differentially targeted by the immune system. Plasma metabolome analysis at age 5 identified 26 metabolites significantly altered in CD progressors, with the microbiota-derived metabolite taurodeoxycholic acid (TDCA) being the most significantly altered one in CD progressors.

Here, we extended our analysis to include samples from the same cohort at age 1 to determine early microbiome alterations. We also used IgA-sequencing to determine the targets of IgA response in the gut microbiome. Furthermore, employing Phylogenetic Investigation of Communities by Reconstruction of Unobserved States (PICRUSt), we identified microbial functions enriched in CD progressors. We previously reported that at ages 2.5 and 5, the gut microbiome of CD progressors has distinct ASV-level differences. Here, we demonstrate that differences in gut microbiome composition are able to be identified as young as 1 year of age, including both ASV-level and among functional metabolic pathways.

## MATERIALS AND METHODS

### Human fecal and plasma samples

The fecal samples were obtained from subjects in the All Babies in Southeast Sweden (ABIS) cohort. ABIS study was ethically approved by the Research Ethics Committees of the Faculty of Health Science at Linköping University, Sweden (Ref. 1997/96287 and 2003/03-092) and the Medical Faculty of Lund University, Sweden (Dnr 99227, Dnr 99321). All children born in southeast Sweden between 1st October 1997 and 1st October 1999 were recruited. Informed consent from the parents was obtained. Fresh fecal samples were collected either at home or at the Well Baby clinic. Samples collected at home were stored at −20°C with freeze clamps, mailed to the Clinical Experimental Research, Department of Biomedical and Clinical Sciences, and stored dry at −80°C. A questionnaire at the 1 year follow up was completed by the parents including, but not limited to, information on breastfeeding duration, antibiotic use, gluten exposure time, and more. We used 10 fecal samples collected for the analysis at age 1 from five individuals who later developed CD and from five healthy controls matched for HLA, breastfeeding, sex, and age.

### Study cohort

All Babies in Southeast Sweden (ABIS) is a prospective population-based study that established a large biobank of biological specimens obtained longitudinally at birth and ages 1, 2.5, and 5. Previously, we reported on gut microbiome differences at ages 2.5 and 5 (34). In total, 17,000 children (78.6%) out of 21,700 born in southeast Sweden 1 October 1997 to 1 October 1999 were included after their parents had given their informed consent. Among this large cohort, 249 children were diagnosed with celiac disease by the end of 2020, validated from the Swedish National Diagnosis Register (SNDR). CD diagnosis was made based on international classification of disease (ICD) codes-10 K90.0 according to the SNDR and met the ESPGHAN CD criteria but detailed information on the height of TTG or any biopsy data with Marsh stage was not available. We used stringent matching criteria including timing of gluten exposure, delivery method, breastfeeding duration, family history of CD, infection history, and use of antibiotics, age, sex, and HLA type (Table S1). We were able to select a sub cohort of 10 individuals, five of which developed CD but were not diagnosed with any other autoimmune disease as of December 2020. We selected these 10 subjects for age 1 because fecal samples obtained from these subjects at ages 2.5 and 5 were analyzed in our previous study (Table S1). The diagnosis of CD for the 5 subjects occurred after the last sample collection at ages 1.8, 6.4, 9.9, 12.6, and 13.2 (Table S1). The diagnosis of CD was confirmed at least twice in accordance with the Swedish National Diagnosis Register (https://doi.org/10.1186/1471-2458-11-450).

### IgA+ and IgA− bacterial separation

IgA-positive (IgA+) and IgA-negative (IgA−) bacteria were separated according to previously established methods onset (34). In summary, frozen human fecal samples were placed in Fast Prep Lysing Matrix D with ceramic beads (MP Biomedicals) and incubated in 1 mL of phosphate-buffered saline (PBS) per 100 mg of samples on ice for 5 minutes to hydrate, followed by homogenization using bead beating for 7 seconds (Minibeadbeater; Biospec). The samples were then centrifuged at 50 *g* for 10 minutes at 4°C to remove large debris. Fecal bacteria in the supernatants were collected (200 µL/sample), washed three times with 500 µL PBS containing 1% (wt/vol) bovine serum albumin (BSA, American Bioanalytical), and then centrifuged for 5 minutes (6,000 × rpm, 4°C). A portion of this washed bacterial suspension (50 µL) was reserved as the pre-sorting sample for 16S sequencing analysis. Following washing, bacterial pellets were resuspended in 50 µL of blocking buffer [PBS containing 1% (wt/vol) BSA and 20% Normal Mouse Serum, Jackson ImmunoResearch] and then incubated for 20 minutes on ice. Subsequently, they were stained with 100 µL of PE-conjugated mouse anti-human IgA (1:40; Miltenyi Biotec clone IS11-8E10) for 30 minutes on ice. After staining, the samples underwent three washes with 500 µL of BSA solution containing 1% (wt/vol) before proceeding to either flow cytometry analysis or cell separation. PE anti-human IgA-stained bacteria were incubated with anti-PE magnetic activated cell orting (MACS) beads (Miltenyi Biotec) (1:5) for 30 minutes on ice and then separated by a custom magnetic plate for 10 minutes on ice. Fecal bacteria bound to the magnetic plate were collected as IgA+ samples for 16S sequencing analysis. Stained and MACS bead-bound bacteria unbound to the magnet were collected (20–40 µL) and passed through MACS molecular columns (Miltenyi Biotec) (one sample/column), followed by flushing with 480 µL PBS containing 1% (wt/vol) BSA. The total pass-through (~500 µL) was loaded onto columns once more. The columns were flushed with 500 µL PBS containing 1% (wt/vol) BSA. The total column pass-through (~1 mL) was saved as IgA− samples for 16S sequencing analysis.

### Fecal IgA flow cytometry analysis

Bacterial cells were extracted from fecal samples following the procedure outlined in the IgA+ and IgA− Bacteria Separation section of this manuscript. These cells were then subjected to staining with PE Anti-human IgA antibodies (1:100; Miltenyi Biotec clone IS11-8E10) for 30 minutes on ice. Following two washes, the bacteria were stained with TO-PRO-3 (ThermoFisher Scientific) to distinguish them from fecal debris or particles. Subsequently, the stained bacteria were analyzed using a BD FACSAriaTM IIIu cell sorter (Becton-Dickinson) according to previously described methods (35), categorizing them as TO-PRO-3 + IgA+/− cells.

### 16S rRNA gene sequencing

16S rRNA sequencing targeting the V4 region was conducted for all bacterial samples using the MiSeq platform, employing barcoded primers, as outlined in previous literature (36). To summarize, bacterial samples were initially suspended in 90 μL of MicroBead Lysis Solution supplemented with 10% RNAse-A and then sonicated in a water bath at 50°C for 5 minutes. Subsequently, the samples were transferred to a plate containing 50 μL of Lysing Matrix B (MP Biomedicals) and homogenized via bead-beating for 5 minutes. Following centrifugation at 4,122 × *g* and 4°C for 6 minutes, the supernatant was carefully transferred to 2 mL deep-well plates (Axygen Scientific). Bacterial DNA from the samples was extracted and purified using the MagAttract Microbial kit (QIAGEN), following the manufacturer’s instructions. PCR amplification of the V4 region of 16S ribosomal RNA was carried out in duplicate (3 µL purified DNA per reaction) with 33 cycles, utilizing Phusion DNA polymerase (New England Bioscience) (36). Subsequently, the amplified PCR products were normalized using the SequalPrepTM normalization plate kit (ThermoFisher Scientific) and then pooled. The concentration of the pooled library was determined using the NGS Library Quantification Complete kit (Roche 07960204001) before being loaded onto a MiSeq sequencer. Illumina MiSeq Reagent Kit V2 (500 cycles) was employed to generate 2 × 250 bp paired-end reads. The raw reads were demultiplexed using Qiime1 (version 1.9), resulting in an average of 30,471 reads per sample.

### Bioinformatic analysis and statistics

Microbial diversity and statistical analyses involved the initial step of filtering and trimming the bacterial 16S rRNA amplicon sequencing reads. Subsequently, sample inference was conducted to convert the amplicon sequences into an Amplicon Sequence Variant (ASV) table using dada2, utilizing the Ribosomal Database Project Training Set 16 (37). Exploratory and inferential analyses were performed by using phyloseq (38) and vegan (39), which includes principle coordinate analysis (PCoA), alpha and beta diversity estimates, and taxa agglomeration. All samples were rarified to a minimum sequencing depth of 20,914 reads using the “rarify_even_depth” function from phyloseq with a random seed set (Fig. S3). Differential OTU abundance was assessed per time point by edgeR (40) with two-sided empirical Bayes quasi-likelihood *F*-tests. *P*-values were corrected by using the Benjamini-Hochberg false discovery rate (FDR), and FDR < 0.1 was considered statistically significant (41). The prediction of gene content and pathway abundance was performed using the Kyoto Encyclopedia of Genes and Genomes (KEGG) database and PICRUSt2 (42–44). Differential KEGG pathway abundance was assessed by using limma (45). The bar plots and box plots were made by using ggplot2 (46) and heatmap by pheatmap (47). An FDR < 0.05 was considered statistically significant.

In our secondary differential abundance analysis based on the reviewer’s suggestion, non-parametric tests were used, including Mann-Whitney *U* tests for unpaired comparisons and Wilcoxon signed-rank tests for paired comparisons. Linear discriminant analysis Effect Size (LEfSe) was employed to identify taxonomic biomarkers between CD progressors and Control groups within each cell type (Presort, IGpos, and IGneg). LEfSe analysis was performed using the default parameters (alpha value for the factorial Kruskal-Wallis test = 0.05, alpha value for the pairwise Wilcoxon test = 0.05, threshold on the logarithmic LDA score = 2.0) (Fig. S2). *P*-values are adjusted using the Benjamini-Hochberg method and an FDR of <0.1 was considered statistically significant. To determine Beta diversity, distance-to-centroid analysis was performed using betadisper and applied PERMANOVA statistics to Bray-Curtis dissimilarity matrices to determine community differences between groups (PCoA). The results of this analysis are reported in Fig. S2.

## RESULTS

### Firmicutes are elevated in the gut microbiome of CD progressors age 1

**Fig 1:**
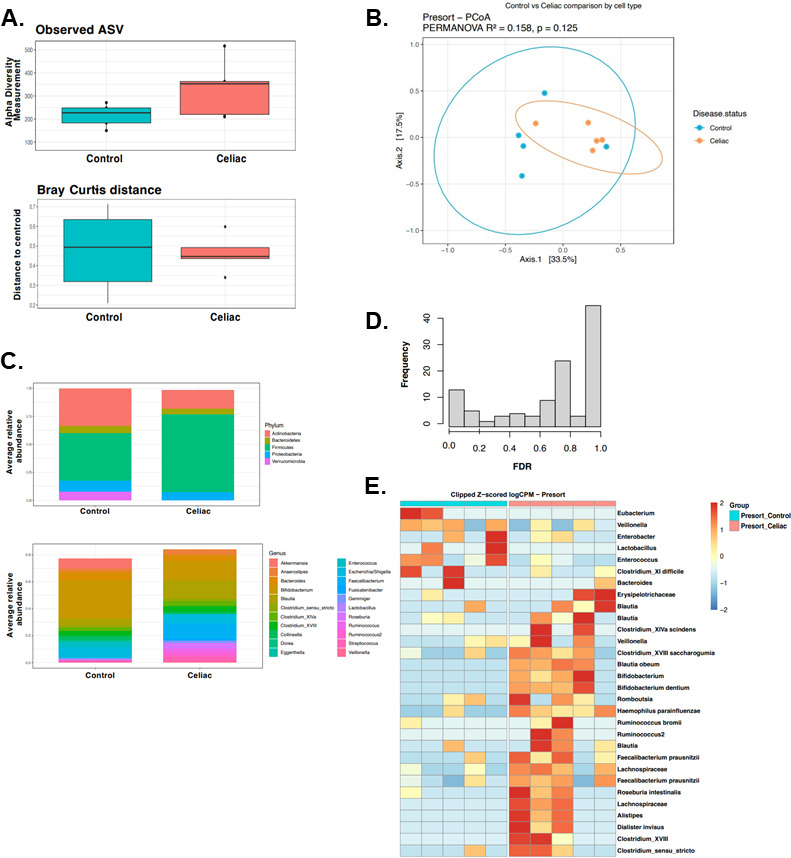
The gut microbiota of CD progressors is distinct at the ASV level. (**A**) Box plots showing the comparison between CD progressors (*n* = 5) and healthy controls (*n* = 5) the alpha diversity measured by observed ASVs (upper panel) and the beta diversity measured by Bray–Curtis dissimilarity (lower panel). Statistical analysis was performed using ANOVA (alpha diversity) and PERMANOVA (Bray-Curtis distance). (**B**) Beta-diversity principal coordinate analysis (PCoA) between CD progressors and healthy controls at age 1 year. Each circle represents an individual CD progressor (orange) or control (blue) sample. Statistical analysis was performed using PERMANOVA. (**C**) Average relative abundance of bacterial phylum (upper panel) or genera (lower panel) of greater than 1% abundance (proportion) between the gut microbiota of CD progressors and healthy controls (taxa average relative abundance >1%). Statistical analysis was performed using Mann-Whitney *U* tests with the Benjamini and Hochberg method to control the false discovery rate (FDR). (**D**) Empirical Bayes quasi-likelihood *F*-tests analysis for the comparisons of gut microbiota ASVs between CD progressors and healthy controls. Frequency: number of ASVs. (**E**) Heat map showing the relative abundance of the top ASVs differentially enriched in CD progressors and healthy controls. Each column represents an individual sample and each row represents an ASV.

In total, we identified 120 amplicon sequencing variants (ASVs) at age 1 (Table S2). While there was a trend of increase in alpha diversity in the CD progressor group (observed ASV: unpaired *t*-test *P* = 0.0887; Simpson index: unpaired *t*-test *P* = 0.0787), consistent with other studies (48), it was not significant (Fig. 1A, upper panel). However, a secondary analysis using a nonparametric statistical approach found a significant increase in the alpha diversity of the CD progressor group compared to healthy controls (Observed: Wilcoxon signed rank test *P* = 0.012) (Fig. S2; Table S7). Beta diversity was comparable between groups in both the original analysis (*P* = 0.915) (Fig. 1A, lower panel) and secondary analysis (*P* = 0.7730057) (Fig. S2). Principal coordinates analysis (PCoA) with PERMANOVA testing showed no significant separation of gut microbiome composition (Fig. 1B). Relative abundance analysis revealed that CD progressors had higher levels of Firmicutes than controls [mean average abundant (MAA): CD = 0.619, Ctrl = 0.427; *P* = 0.0148] (Fig. 1C, upper panel, Table S3). On the other hand, there was no difference in other phylogenetic levels including at the genus level (Fig. 1C, lower panel). Focusing on ASV-level differences, we found only 14 ASVs that differed at age 1 (Fig. 1E; Table S2), with the majority remaining unchanged (FDR < 0.1, *P* < 0.05; Fig. 1D). For example, *Ruminococcus bromii* (FC = 3,410, FDR = 0.05), *Dialister invisus* (FC = 1,410, FDR = 0.05), and *Bifidobacterium dentium* (FC = 1,060, FDR = 0.05) were enriched in CD progressors along with some ASVs from the *Clostridium* genus including Clostridium XVII (FC = 4,560, FDR = 0.05) and *Clostridium XIV scindens* (FC = 309, FDR = 0.05). Interestingly, most of these *Clostridium* species are related to TDCA production (34) that we recently identified a potential role in CD onset (34). Conversely, Enterococcus (FC = −1,000, FDR = 0.05) was enriched in control samples (Fig. S1; Fig. 1D and E). However, it is noteworthy that there was considerable variability in the occurrence of certain ASVs within both groups. Additionally, secondary analysis using a nonparametric statistical approach on the same data identified no significant differences between the control and the CD progressor groups at any phylogenetic level (Table S8 and S9).

### CD progressors IgA response at age 1

**Fig 2:**
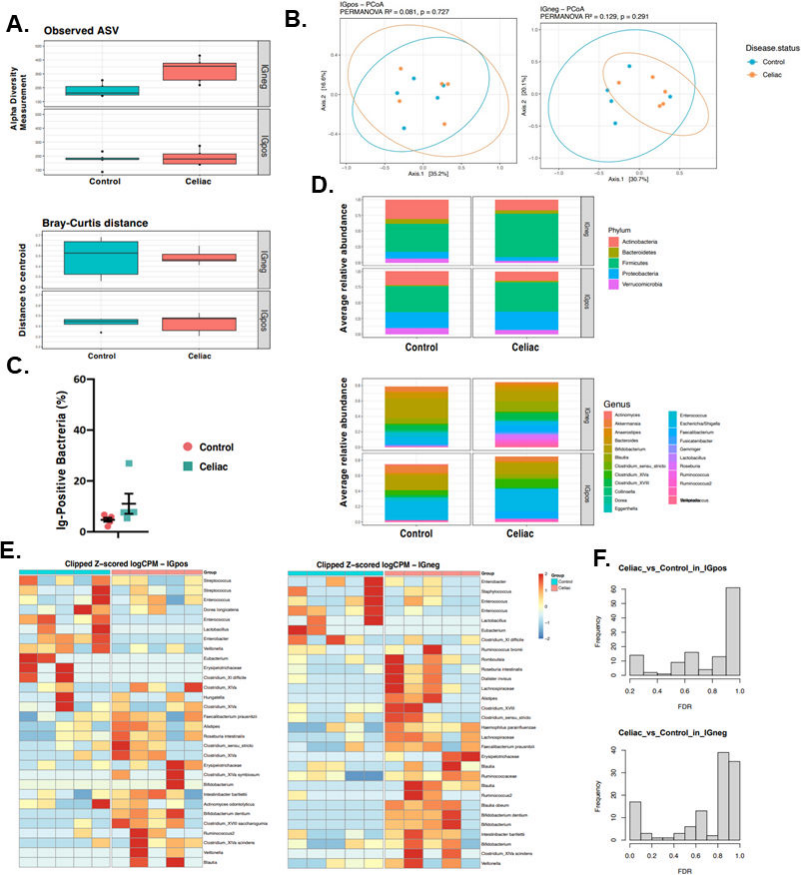
IgA-based sorting and 16S sequencing revealed that gut microbiota were differentially coated by IgA. (**A**) Box plots showing the comparison between CD progressors and healthy controls: the alpha diversity measured by observed for IgA+/IgA− microbiota (upper panel), and the beta diversity measured by Bray–Curtis dissimilarity for IgA+/IgA− microbiota (lower panel) at age 1 for CD progressors (orange) and healthy controls (blue). Statistical analysis was performed using ANOVA (alpha diversity) and PERMANOVA (Bray-Curtis distance). (**B**) Beta diversity principal coordinate analysis (PCoA) of sample similarity/dissimilarity between IgA+ (left) and IgA− (right) microbiota in healthy control (blue) or CD progressors (orange). Each point represents an individual sample. Statistical analysis was performed using PERMANOVA. (**C**) Percentage of IgA positive bacteria recovered (blue) and healthy controls (red). Indicated are mean ± SEM. Statistical analysis was performed using two-way ANOVA (**D**) Average relative abundance of IgA+/IgA− bacterial phylum (upper) or genera (lower) of greater than 1% abundance (proportion) between the gut microbiota of CD progressors and healthy controls (taxa average relative abundance >1%). Statistical analysis was performed using Mann-Whitney *U* tests with Benjamini and Hochberg method to control false discovery rate (FDR). (**E**) Heat map showing the relative abundance of the top ASVs significantly different between IgA+ (left) IgA− (right) CD progressors (red) and healthy controls (blue). Each column represents an individual sample and each raw represents an ASV. (**F**) Empirical Bayes quasi-likelihood *F*-tests analysis for the comparisons of IgA-coated (top panel) or non-coated (bottom panel) gut microbiota ASVs between CD progressors and healthy controls.

To identify microbiota species highly coated with IgA and determine whether there is any difference in the IgA response at age 1, we used a modified method of IgA-sequencing (34). In the IgA− group, the alpha diversity was higher (observed ASV: unpaired *t*-test *P* = 0.0121; Simpson index: unpaired *t*-test *P* = 0.0828) for the CD progressors compared to the controls (Fig. 2A, upper panel), while the alpha diversity of the IgA+ fractions was similar (Fig. 2A, upper panel). These results were similar to those of our secondary analysis, where we also found that the IgA− CD progressor group had a higher alpha diversity compared to the controls (Observed: Wilcoxon signed rank test *P* = 0.012186) (Fig. S2). Beta diversity was comparable in all groups in both primary (Fig. 2A, lower panel) and secondary analysis (Fig. S2). PCoA analysis showed no significant separation between IgA+ and IgA− bacteria both in control and CD samples (Fig. 2B). IgA+ bacteria accounted for 4.57% (LS mean) at age 1 in controls (Fig. 2C) and 10.99% at age 1 in CD progressors though this difference was not statistically significant (*P* = 0.24). Consistent with the pre-sorting data, CD progressors had higher levels of Firmicutes than controls for both IgA+ and IgA− fractions (for IgA+ MAA: CD = 0.4702, Ctrl = 0.4097; *P* = 0.0422 and for IgA− MAA: CD = 0.6898; Ctrl = 0.4097; *P* = 0.0171) (Fig. 2D, upper panel, Table S2); however, in our secondary analysis, this difference was not identified (Table S8). We did not observe any differences at other phylogenetic levels in either analysis (Fig. 2D, lower panel, Tables S3 and S9).

Notably, we did not identify any differences between IgA− and IgA+ fractions of controls or CD progressors. There was also no difference between the IgA+ fractions of both groups. However, several taxa were differentially abundant (FDR < 0.1) between CD progressors and healthy controls in the IgA-negative microbiota (Fig. 2E and F) following our observations in the basal microbiome differences. Erysipelotrichacea (FC = 2,170, *P* = 0.00438, FDR = 0.0739), Ruminococcus2 (FC = 1,800, *P* = 0.0103, FDR = 0.0775), *B. dentium* (FC = 1,720, *P* = 0.00332, FDR = 0.0739), *Roseburia intestinalis* (FC = 1,670, *P* = 0.00517, FDR = 0.0739), and Clostridium_XVIII (FC = 1,310, *P* = 0.00888, FDR = 0.0761) were enriched in the IgA− microbiota of CD progressors compared to healthy controls. In contrast, three taxa were decreased in the IgA− gut microbiota of CD progressors including Enterococcus (FC = −2,900, *P* = 0.00323, FDR = 0.0739), Lactobacillus (FC = −1,410, *P* = 0.0069, FDR = 0.0739), and Eubacterium (FC = −809, *P* = 0.00606, FDR = 0.0739) (Table S2).

### Pathogenesis and inflammation-related functions are enriched in CD progressors’ gut microbiota

**Fig 3:**
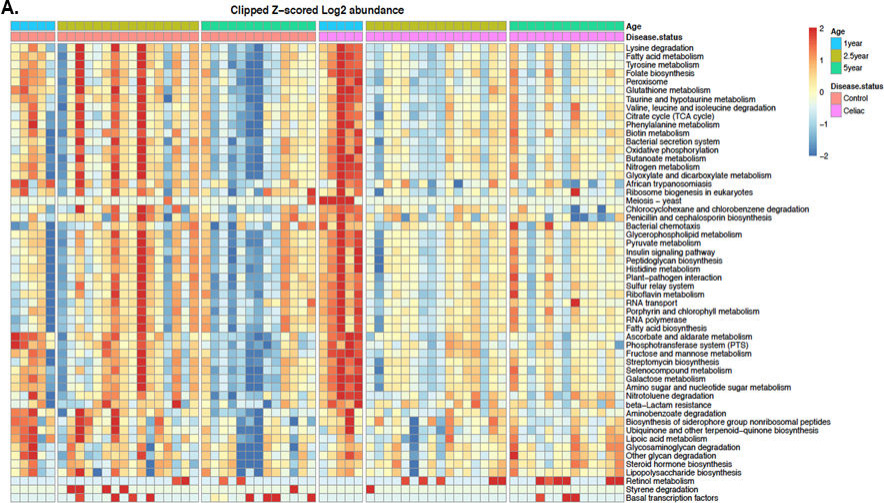
The enriched microbial pathways altered in CD progressors’ gut microbiota are significantly different from healthy controls. Heat map of PICRUSt predicted metabolic pathways of CD progressors and healthy controls. Each column represents an individual participant, and each row represents a predicted microbial functional pathway. Color code is shown on the figure. Ages 1 year old: *n* = 5/group; age 2.5 years old: *n* = 16/group; age 5 years old: *n* = 13/group.

PICRUSt analysis (35) is designed to estimate the functional metagenome of gut bacteria using 16S rRNA data. In this study, we utilized PICRUSt to examine enriched functional pathways in CD progressors. To this end, we combined the sequencing data from CD progressors at age 1 in this study and ages 2.5 and 5 reported in our previous study (34), noting that all sample preparation and sequencing were conducted simultaneously for consistent results. Combining PICRUSt with Kyoto Encyclopedia of Genes and Genomes (KEGG) metabolic pathway analysis, we identified 71 different metabolic pathways that differed between CD and control samples at age 1 (FDR < 0.05, Fig. 3,; Table S5). Among these pathways, N-glycan biosynthesis (FC = 3.42, FDR = 0.0208), penicillin and cephalosporin biosynthesis (FC = 2.75, FDR = 0.0259), beta-lactam resistance (FC = 2.51, FDR = 0.0208), and bacterial chemotaxis (FC = 2.28, FDR = 6.47e-4) pathways were among the top pathways enriched in CD progressors. Interestingly, most of these pathways are involved in bacterial pathogenesis (36, 49) or shaping the composition of microbiota (50, 51). Additional pathways that were highly enriched at age 1 included the fatty acid biosynthesis pathway (FC = 1.92, *P* = 0.000217, FDR = 0.0082), pathways related to D-glutamine and D-glutamate metabolism (FC = 1.66, *P* = 0.00397, FDR = 0.0208), nitrogen metabolism (FC = 1.6, *P* = 0.0354, FDR = 0.0082), glyoxylate and dicarboxylate metabolism (FC = 1.92, *P* = 0.00513, FDR = 0.0209), glycine, serine, and threonine metabolism (FC = 1.51 *P* = 0.0122, FDR = 0.0296). We also identified nine pathways showing trends of difference both at age 2.5 and at age 5 (Table S5). For example, styrene degradation, lysine degradation, fatty acid metabolism, and glutathione metabolism were decreased in CD progressors at age 2.5 (*P* < 0.05). Meanwhile, retinol metabolism, steroid hormone biosynthesis, and glycosaminoglycan degradation pathways were increased in CD progressors at age 5 (*P* < 0.05). However, when we applied a secondary statistical analysis involving a nonparametric approach, we were unable to identify a similar statistically significant difference between the CD progressor and the control groups’ functional pathways at any age (Fig. S4; Table S10).

**Fig 4:**
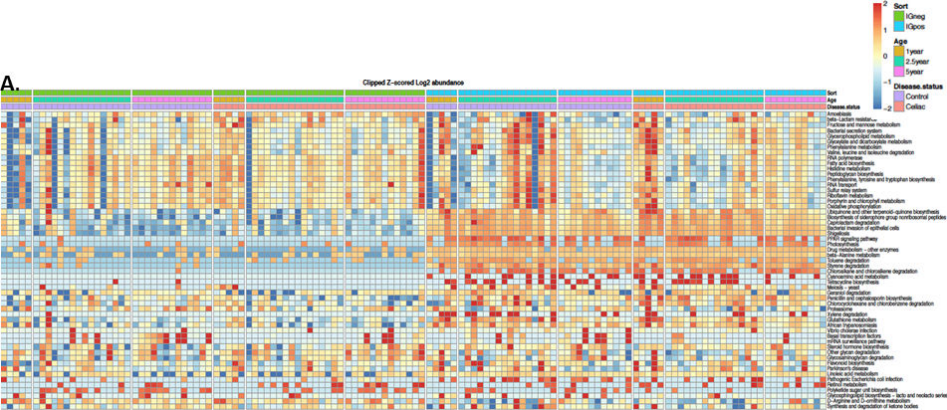
The enriched microbial pathways altered in CD progressors’ gut microbiota are significantly different between IgA+ and IgA− bacteria. Heat map of predicted metabolic pathways of CD progressors and healthy controls obtained from PICRUSt analysis after IgA sequencing. Each column represents an individual participant, and each row represents a predicted microbial functional pathway. Color code is shown on the figure. Age 1 years old : *n* = 5/group; age 2.5 years old: *n* = 16/group; age 5 years old: *n* = 13/group.

We also used PICRUSt to examine the functional pathways comparing IgA+ to IgA− microbiota (Fig. 4; Fig. S3; Table S5). We identified styrene degradation pathway enriched in IgA+ population at all ages. Furthermore, we identified 31 different functional pathways at age 2.5 and 23 functional pathways at age 5 (FDR < 0.05) in the healthy subjects. The top enriched pathways in healthy IgA+ population were styrene degradation (age 2.5: FC = 103, FDR = 1.97e−12; age 5: FC = 77.1, FDR = 4.92e−9), chloroalkane and chloroalkene degradation (age 2.5: FC = 534, FDR = 9.09e−11; age 5: FC = 222, FDR = 1.42e−6), and toluene degradation (age 2.5: FC = 482, FDR = 2.3e−7; age 5: FC = 99.6, FDR = 1.73e−3). Analyzing CD samples, we identified 7 pathways at age 1, 28 pathways at age 2.5, and 13 pathways at age 5 enriched in IgA+ microbiota (FDR < 0.05). The most significantly enriched pathways in CD progressors’ IgA+ microbiota were beta-alanine metabolism (age 1: FC = 1,210, FDR = 0.033; age 2.5: FC = 145, FDR = 7.43e−4; age 5: FDR > 0.05), chloroalkane and chloroalkene degradation (age 1: FC = 997, FDR = 4.86e−5, age 2.5: FC = 148, FDR = 1.83e−7; age 5: FC = 714, FDR = 2.44e−8), and styrene degradation (age 1: FC = 856, FDR = 1.87e−8, age 2.5: FC = 421, FDR = 1.42e−19; age 5: FC = 440, FDR = 8.43e−15). At age 1, CD progressors had more metabolic pathways predicted to be enriched in the IgA+ samples compared to healthy control. For example, pathogenic pathways, bacterial invasion of epithelial cells (FDR = 0.0174, FC = 16.2), and beta-alanine metabolism (FDR = 0.033, FC = 1210) were identified in CD IgA+ microbiota population but were absent in control IgA+ at age 1. Our secondary analysis with using a nonparametric statistical approach identified 74 pathways at age 5 and 21 pathways at age 2.5 that were enriched in the healthy control group’s IgA+ microbiota. Furthermore, we identified 13 pathways at age 5 and 34 pathways at age 2.5 that were enriched in CD progressor’s IgA+ microbiota. (Fig. S5; Table S10). Most of the functional pathways identified with the secondary nonparametric analysis in both of these groups are the same pathways identified in our primary analysis (Fig. 3).

## DISCUSSION

Recent studies have demonstrated strong associations between the gut microbiota and the pathogenesis of autoimmune diseases including Type 1 diabetes (52, 53), multiple sclerosis (54, 55), and rheumatoid arthritis (56, 57). Studies of the gut microbiome in CD have demonstrated intestinal dysbiosis in CD patients (20, 22–27, 58, 59). However, most of these studies utilized adult samples from individuals already diagnosed with the disease rather than adopting a longitudinal, prospective approach with samples collected prior to disease onset, as performed in this study. Human gut microbiota development is divided into three phases: a developmental phase (months 3–14), a transitional phase (months 15–30), and a stable phase (months 31–46) (60). Because recent data show that many childhood CD cases will develop in the first years of life (61), we analyzed samples representing all of these critical phases. We previously reported that CD progressors at ages 2.5 and 5 have a distinct gut microbiome composition and differential IgA response and at age 5 have an altered cytokine profile and plasma metabolome. Here, we report, for what we believe is the first time, that there are significant differences in the predicted functional levels at the developmental phase in CD progressors’ gut microbiome. Consistent with previous reports (59), we identified the proportion of the phylum Firmicutes to be higher in CD progressors at age 1 (Fig. 1C). Bacterial proteases of species mostly classified within the Firmicutes phylum are involved in gluten metabolism and this might be a potential link to CD (62). Additionally, we identified bacterial species enriched in CD progressors at age 1 including *Bifidobacterium dentium*, *Clostridium XIVa sciendens*, *Lachnospiraceae,* and *Alistipes,* and a trend of increase in *Clostridium sensu stricto* and *Faecalbacterium prausnitzii*; however, there was variation between samples. This is consistent with previous research findings that show an increase of *Faecalbacterium prausnitzii* in untreated CD (63), *Lachnospiraceae* in CD progressors (30), and infants at high risk of developing the disease (64). In our previously published paper using the same cohort, we found that *Lachnospiraceae* continue to be enriched at ages 2.5 and 5 in CD progressors (34). Additionally, *Alistipes* was increased in CD patients (63, 65). Notably, a previous study reported that the abundance of *R. bromii* was greatly reduced in CD patients when gluten-free (GF) diet was introduced (66). Furthermore, infants at high risk for developing CD had higher levels of *Clostridium sensu stricto* (67). We found that several taxa were decreased in the age 1 CD progressor samples, such as *Enterococcus, Lactobacillus, and Eubacterium*. Our previous study involving samples from the same donors found that there was a decrease in *Eubacterium* species at ages 2.5 and 5 years (34). Consistent with our findings, other studies have shown that the abundance of *B. dentium* was increased in the CD patients (68). *Clostridium XIVa* genera is responsible for producing the proinflammatory metabolite TDCA, which might be implicated in CD pathogenesis. In our previous study, we showed that TDCA was enriched in serum of CD progressors at age 5 (34). In this study, as well as in our previous study, we showed that several Clostridium members that are potentially related with TDCA levels were enriched in CD progressors. When we compared our data in this study (age 1) to the data obtained from our previous study (ages 2.5 and 5), we found that the differences in the gut microbiota were less drastic at the phylum level but significantly increased in the ASV level by age.

Intestinal IgA plays a crucial role in defending against pathogenic microorganisms and in maintaining gut microbiome homeostasis. Interestingly, IgA-deficient patients are more susceptible to a variety of pathologies, including CD (69). Planer et al. described mucosal IgA responses progression during two postnatal years in healthy US twins (70). They showed that (i) IgA-coated bacteria is affected by age and host genetics and (ii) IgA response is determined by “intrinsic” properties of gut microbiota community members. We used a similar approach to investigate the gut immune development toward healthy and CD states, and we initially focused on the development of the IgA response during the gut microbiota maturation. At age 1, we did not identify any ASVs that were significantly different in IgA− and IgA+ samples. While this is potentially related to the small sample size used in this study, it might also suggest that the intestinal IgA response is not mature enough to target specific bacteria in the gut. However, we showed that the IgA response is highly selective, and only a small fraction of the gut microbiota is highly coated with IgA in the first year of life. This is consistent with our previous findings at ages 2.5 and 5, and notably, we found a significant increase in the percentage of IgA-coated bacteria in CD progressors at age 5 (34). While a reduction of secretory IgA (sIgA) using infant (4–6 months) fecal samples in CD progressors (27) was reported previously, we did not identify any significant difference in the ratio of IgA-coated bacteria at age 1.

The abundance analysis (Table S2; Fig. 2E) showed that CD progressors’ IgA+ microbiota is enriched with Firmicutes. Consistent with this finding, previous studies reported higher proportions of Firmicutes in HLA high-risk infants (29, 67). Notably, most of the bacterial strains in the human gut microbiota that can metabolize gluten are classified with the Firmicutes, which includes strains that have been found to show extracellular proteolytic activity against gluten proteins (71). Bacterial metabolism of gluten might result in different gluten peptide fragments, potentially related to CD autoimmunity.

PICRUSt analyses showed significant differences at all developmental phases, in particular, within the transition period at age 1. Most significant differences were identified in pathways related to bacterial pathogenesis and shaping the composition of microbiota. For example, glutathione metabolism was greatly decreased in CD progressors. Decreased glutathione redox cycle in CD patients is strongly associated with disease development (72). Several other pathways found enriched in age 1 samples have previously been found to be upregulated in CD patients (73), including pathways related to D-glutamine and D-glutamate metabolism, nitrogen metabolism, glyoxylate and dicarboxylate metabolism, glycine, serine, and threonine metabolism. An overproduction of nitric oxide (NO), a product of nitrogen metabolism, has been associated with the disruption of gut microbiota and directly linked with autoimmune conditions involving gut inflammation, such as Crohn’s disease and ulcerative colitis (74, 75). The fatty acid biosynthesis pathway was significantly increased at age 1. Alterations in the levels of fatty acids are associated with gastrointestinal diseases such as CD (76). Fatty acids can modulate the function of immune cells and inflammatory processes both directly and indirectly (77). In particular, short-chain fatty acids (SCFAs) are metabolites produced by the gut microbiota and play a role in gut barrier maintenance. The alteration and accumulation of some SCFAs may be associated with untreated CD (78). At age 5, PICRUSt predicted a trend that retinol metabolism, steroid hormone biosynthesis, and glycosaminoglycan degradation as over-represented pathways in CD progressors. Retinoic acid is one of the products of retinol metabolism and plays a key role in the intestinal inflammatory response (79) as well as impacting the expression of cytokine expression (80). A previous study showed that retinoic acid-mediated inflammatory responses to gluten in both *in vitro* and *in vivo* studies (81). The increased retinol metabolism and glycosaminoglycan degradation pathways in CD progressors are potentially related to chronic inflammation. Indeed, glycosaminoglycan helps form a protective barrier for the intestinal mucin. The breakdown of glycosaminoglycan is reported to be associated with inflammatory response in intestinal disorders such as IBD (82).

We also used PICRUSt after sorting via IgA sequencing to compare the functional pathways associated with the IgA+ and IgA− bacteria. Among our CD progressor samples, we identified 7 pathways at age 1, 28 pathways at age 2.5, and 13 pathways at age 5 enriched in IgA+ microbiota. Among these were a pathway involved in the bacterial invasion of epithelial cells, which was predicted in the IgA+ microbiota, but not the IgA− microbiota at age 1.

The gut microbiome develops in the first years of life by progressing through several distinct phases. Entering the transition phase, the gut microbiota in CD progressors displayed more proinflammatory and oxidative stress-related features. At stable phases, gut microbiota in CD progressors began to become more involved in functions related to the clinical manifestation of the disease, such as those associated with intestinal inflammation. This longitudinal observation provides insight into the proinflammatory and pathogenic function of gut microbiota in different stages of early CD pathogenesis.

Currently, the only way to treat CD is strict adherence to a gluten-free (GF) diet, but 20% of patients do not respond to GF diet and continue to have persistent or recurrent symptoms (10). CD permanently reshapes intestinal immunity and alterations in TCRγδ + intraepithelial lymphocytes in particular may underlie non-responsiveness to the GF diet (83). Our findings suggest that enriched pathways identified in the CD progressors’ gut microbiota might be related or contribute to an inflammatory environment that could be an important component of intestinal inflammation in CD. The pro-inflammatory pathways identified in this study could potentially trigger local and systemic inflammation independent of the diet and may explain a failure to respond to GF diet in some patients.

The main strength of this study lies in the longitudinal sampling that represents all three phases of gut microbiota development in children for the PICRUST analysis. Furthermore, applying IgA-seq analysis, for this age group, we examined an important dimension to evaluate CD pathogenesis. On the other hand, there are several limitations. The most important limitation is the small sample size, particularly at age 1 (*n* = 5), which is due to stringent and robust matching criteria between CD progressor and control groups. A total of 249 subjects were diagnosed with CD in the ABIS cohort, but samples were not available from all of them. After matching these subjects to healthy controls using stringent criteria—such as age, sex, HLA type, time of gluten exposure, delivery method, breastfeeding duration, infection history, and antibiotic use—we were limited to selecting only 16 CD progressors for inclusion in our study. One of the participants was diagnosed with CD at age 1.8, and because the number of samples was limited, this subject’s 2.5 year sample was still analyzed in the PiCRUST analysis. Therefore, this study should be considered a pilot study indicating important differences with a small sample size; however, the analysis should be repeated with much larger sample sizes to draw more generalizable conclusions. Using 16S-sequencing is another limitation that requires PICRUST to predict functional pathways instead of shotgun sequencing directly acquiring the gene information. Therefore, follow-up studies with shotgun sequencing are needed to validate our findings, increasing the statistical power and depth.

Variability in data from microbiome studies can vary widely depending on the bioinformatics pipelines and analysis methods used. We are publishing our results obtained from using parametric statistical methods; however, we are also reporting supplemental figures and metadata obtained from our secondary analysis using non-parametric statistical analysis methods. We have used EdgeR for differential abundance analysis and identified 14 ASVs that are differentially abundant in our age 1 samples. Because EdgeR uses a parametric model that, in combination with our small sample size may have identified false positives, we reexamined our statistical approach using non-parametric (Mann-Whitney *U* tests for unpaired comparisons and Wilcoxon signed-rank tests for paired comparisons) testing. Using this approach, we were unable to identify any statistically significant differences in the abundance of ASVs present in our age 1 samples (Fig. S2; Table S8). We also did not identify any statistical differences at other phylogenetic levels (Table S9). Alpha and beta diversity was reanalyzed, and the results for beta diversity remained unchanged. However, when a nonparametric statistical approach was applied to alpha diversity, we discovered a significant increase in the alpha diversity in CD progressors compared to healthy controls (Fig. S2; Table S7). In our new analysis, we applied LEftsE analysis to identify features that could be responsible for differences between CD progressor and control groups. While this could potentially highlight taxonomic biomarkers, all identified features have FDR-adjusted *P*-values of >0.1 (Fig. S2; Table S11). Our assessment of functional pathways using PICRUST analysis was also repeated using a nonparametric statistical approach. For the presorted data (Fig. S4; Table S10), we were unable to identify any significant differences in our secondary analysis. For the post-sorted data, many of the identified enriched pathways are consistent with our primary analysis (Fig. S5; Table S10)

In conclusion, taken together, our findings suggest that the gut microbiota of CD progressors in the first 5 years of life has functionally different gut microbiota and this can potentially contribute to the onset and progression of CD. We identified a small number of ASVs at age 1 that were differentially present in the CD progressors microbiota, some of which have been previously reported to be associated with those at high risk for developing CD or patients in active CD. We also identified several functional pathways enriched in CD progressors at each stage of microbiome development that are involved in bacterial pathogenesis, shaping the microbiota composition, and gut inflammation, as well as some that have been previously linked to CD. Understanding the role of the gut microbiota in chronic inflammation in CD and targeting inflammatory bacteria or developing anti-inflammatory probiotics/prebiotics may open novel approaches to understand disease pathogenesis and reveal new preventive and treatment models.

## Data Availability

The 16S raw sequencing data generated in this study are available at NCBI Sequence Read Archive Bioproject PRJNA631001. Table S6 contains the sample number to SRA association. Disease status of each subject is found in Table S1. The gut microbiome analysis codes generated in this study are available at https://github.com/altindislab/celiac_disease_age1

## SUPPLEMENTAL MATERIAL

Supplemental figures spectrum.01468-24-s0001.pdf Figures S1 to S5.

Table S1 spectrum.01468-24-s0002.xlsx Detailed cohort and fecal sample information.

Table S2 spectrum.01468-24-s0003.xlsx ASV statistics.

Table S3 spectrum.01468-24-s0004.xlsx Relative abundances at phylum, class, order, family, and genus.

Table S4 spectrum.01468-24-s0005.xlsx Top ASV and metabolic pathway Pearson correlation.

Table S5 spectrum.01468-24-s0006.xlsx PICRUSt analysis.

Table S6 spectrum.01468-24-s0007.xlsx Sample ID/SRA association, diversity statistics, ASV statistics, LEfSe statistics, and PICRUSt analysis (nonparametric statistical approach),

Table S9 spectrum.01468-24-s0008.xlsx Relative abundances at phylum, class, order, family, and genus level.

ASM does not own the copyrights to Supplemental Material that may be linked to, or accessed through, an article. The authors have granted ASM a non-exclusive, world-wide license to publish the Supplemental Material files. Please contact the corresponding author directly for reuse.
