# Supplementary material for: Children who develop celiac disease are predicted to exhibit distinct metabolic pathways among their gut microbiota years before diagnosis
Source: Microbiol Spectr. 2025 Feb 4;13(3):e01468-24. doi: 10.1128/spectrum.01468-24 (PMC11878042; doi:10.1128/spectrum.01468-24)
Supplement: Supplemental figures — Figures S1 to S5. [file spectrum.01468-24-s0001.pdf]

Figure S1: Violin plot representation of most enriched genus/species in CD progressors compared to healthy controls at age 1

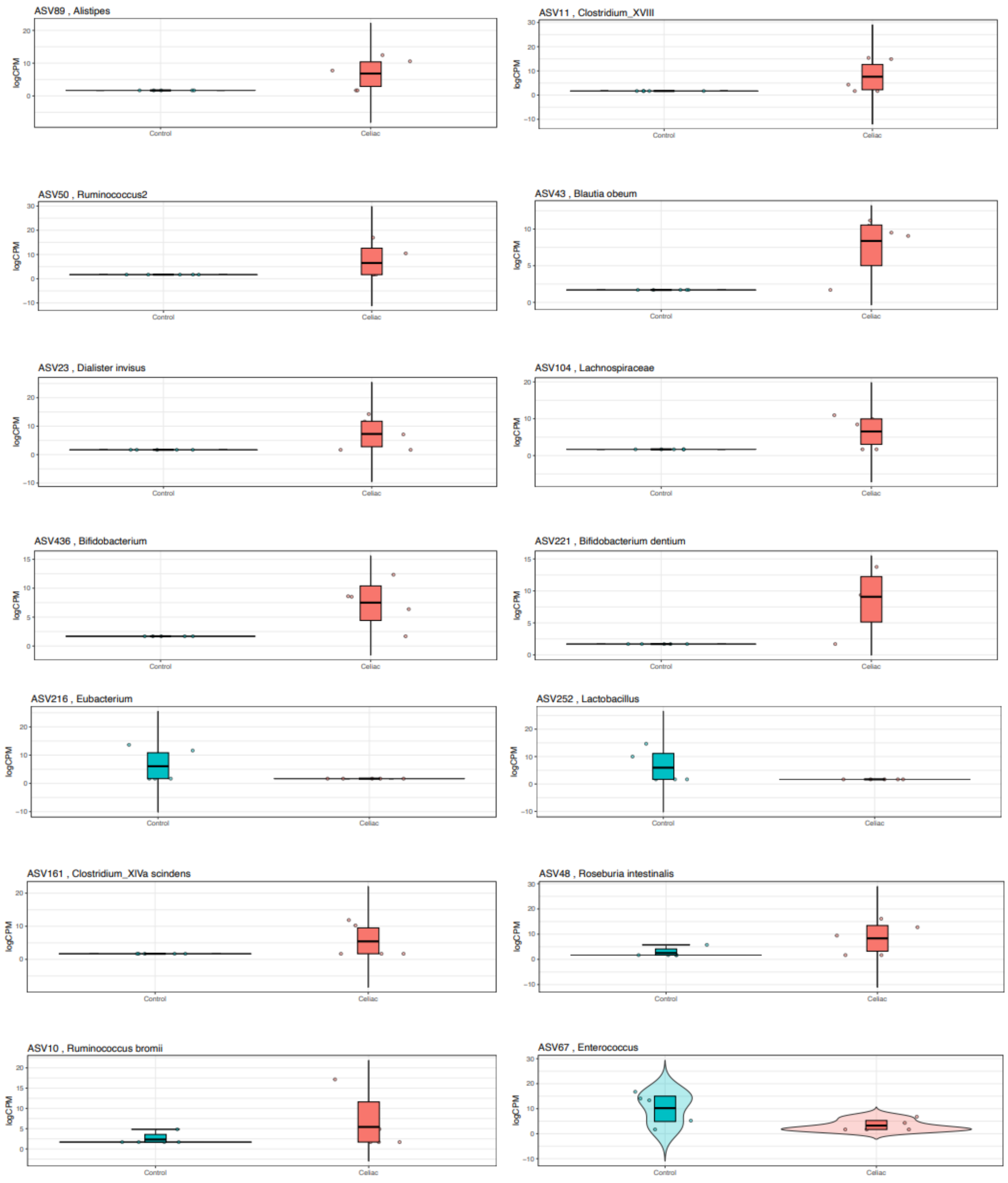

Figure S2: Secondary analysis of data using a nonparametric approach.

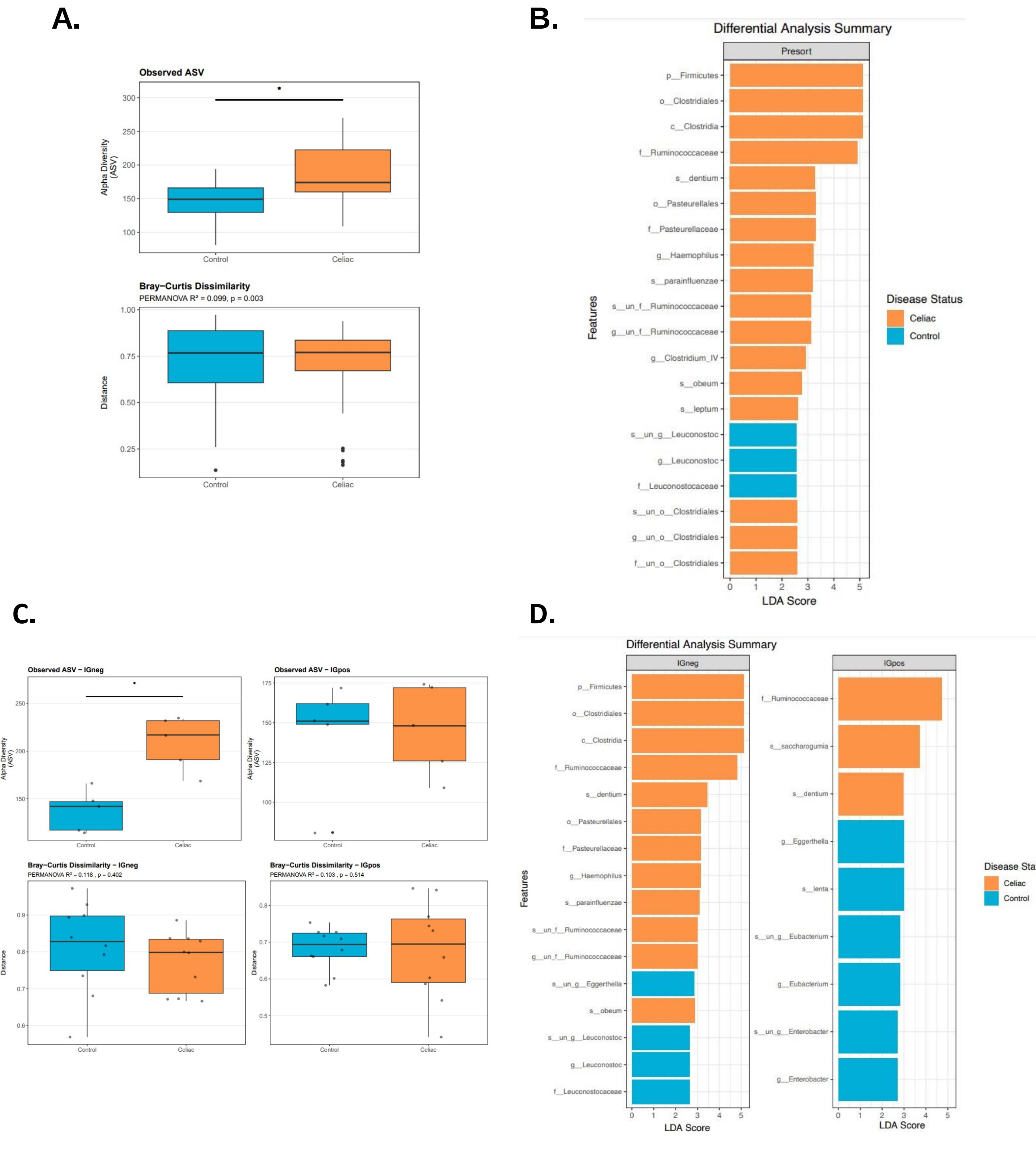

Figure S3: Rarefaction of samples

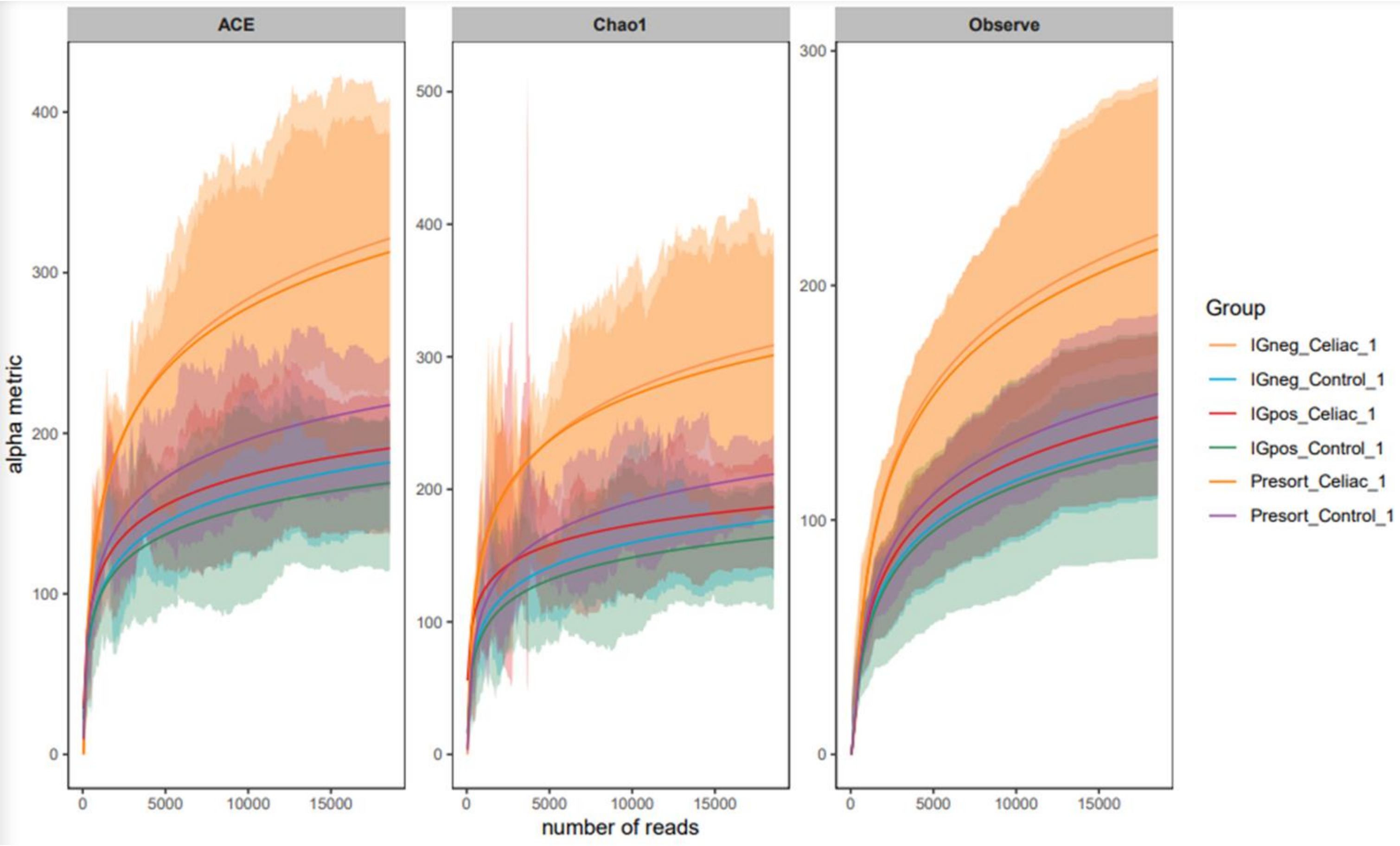

Figure S4: PICRUST analysis- the enriched microbial pathways altered in CD progressors' gut microbiota are not significantly different from healthy controls with secondary nonparametric statistical approach

A.

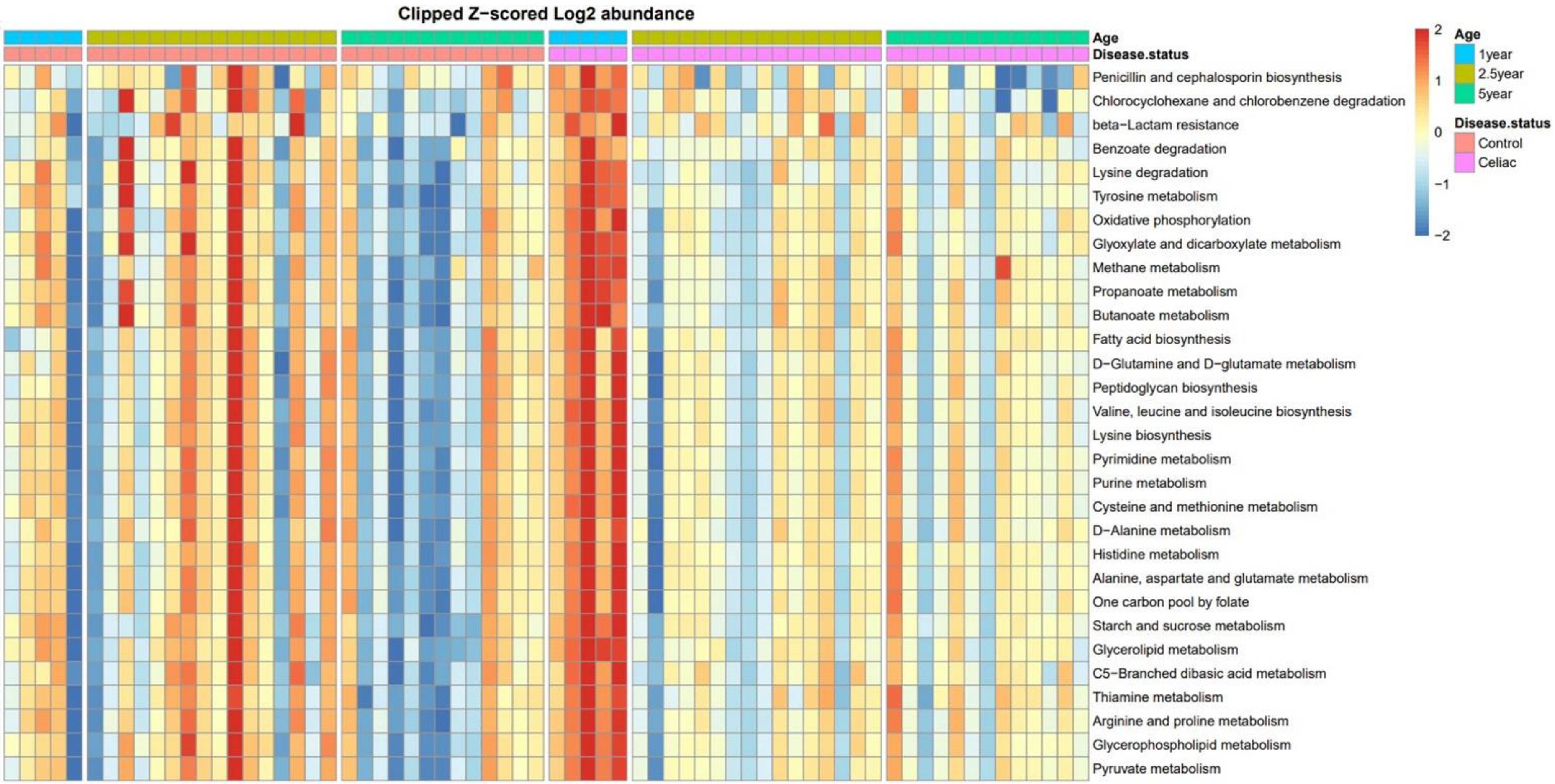

Figure S5: The microbial pathways in CD progressors' gut microbiota are not significantly different between IgA+ and IgA- bacteria with secondary nonparametric statistical approach

A.

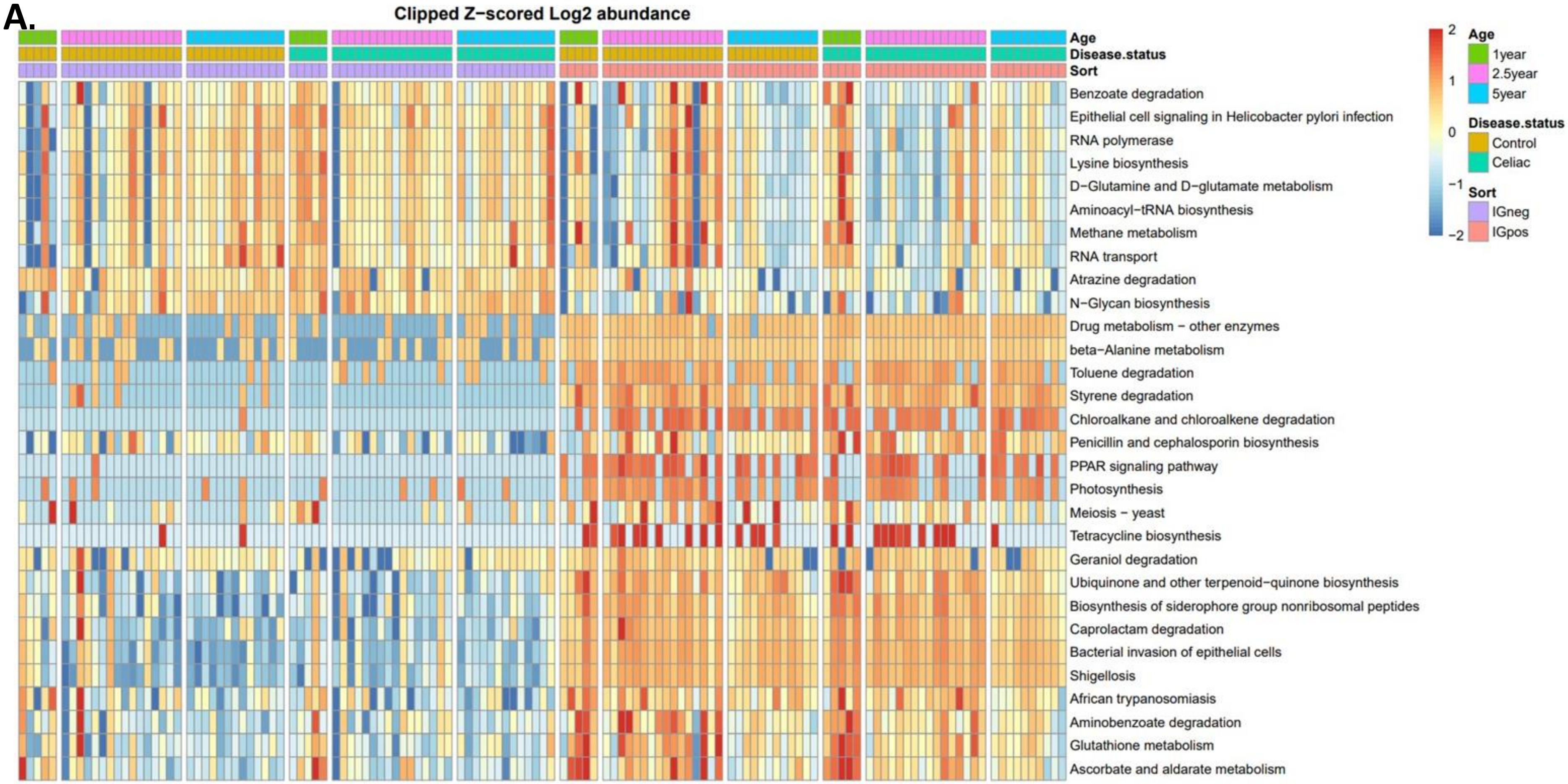

Supplementary Figure Legends

Figure S1. Violin plot representation of most enriched genus/species in CD progressors compared to healthy controls.

Violin plots showing the abundance of ASVs in CD progressors (orange, n=5) and healthy controls (blue, n=5) with False Discovery Rate less than 0.1. Statistical significance was determined using empirical Bayes quasi-likelihood F-tests.

Figure S2: Secondary analysis of data using a nonparametric approach.

A. Box plots showing the comparison between CD progressors (n=5) and healthy controls (n=5) the alpha diversity measured by observed ASVs (upper panel) and the beta diversity measured by Bray–Curtis dissimilarity (lower panel). Statistical analysis was performed using Mann-Whitney (alpha diversity) and PERMANOVA (Bray-Curtis distance).

B. LEfSe analysis of taxonomic distribution in presort gut microbiota between CD progressor and control groups at age 1. Linear discriminant analysis (LDA) Effect Size (LEfSe) showing differential abundance scores for taxa between CD progressors and healthy controls in presorted gut microbiota samples at age 1. Orange bars represent taxa with higher LDA scores in CD progressors, while blue bars represent taxa with higher scores in controls. The length of each bar represents the LDA score, indicating the magnitude of the abundance difference. Taxa are labeled with their taxonomic classification (p: phylum, c: class, o: order, f: family, g: genus, s: species, un: unclassified).

C. Box plots showing the comparison between CD progressors and healthy controls: the alpha diversity measured by observed for IgA+/IgA- microbiota (upper panel), and the beta diversity

measured by Bray–Curtis dissimilarity for IgA+/IgA- microbiota (lower panel) at age 1 for CD progressors (red) and healthy controls (blue). Statistical analysis was performed using Mann-Whitney (alpha diversity) and PERMANOVA (Bray-Curtis distance).

D. LEfSe analysis of taxonomic distribution in presort gut microbiota between CD progressor and control groups at age 1. LEfSe analysis showing differential abundance scores for taxa between CD progressors and healthy controls in presorted gut microbiota samples at age 1. Orange bars represent taxa with higher LDA scores in CD progressors, while blue bars represent taxa with higher scores in controls. The length of each bar represents the LDA score, indicating the magnitude of the abundance difference. Taxa are labeled with their taxonomic classification (p: phylum, c: class, o: order, f: family, g: genus, s: species, un: unclassified).

Figure S3: **Rarefaction of Samples**

Alpha diversity rarefaction curves comparing CD progressor and control groups across cell types. Rarefaction curves showing three alpha diversity metrics (ACE, Chao1, and Observed) for gut microbiota samples from CD progressor and healthy controls at age 1. Samples are grouped by cell type (Presort, IGpos, and IGneg) and disease status (Celiac vs Control). Lines represent the mean diversity values, while shaded areas indicate the 95% confidence intervals. The x-axis shows sequencing depth (number of reads), and the y-axis shows the alpha diversity metric values.

Figure S4: The enriched microbial pathways altered in CD progressors' gut microbiota are not significantly different from healthy controls with secondary nonparametric statistical approach

Heat map of PICRUSt predicted metabolic pathways of CD progressors and healthy controls. Each column represents an individual participant and each row represents a predicted microbial

functional pathway. Color code is shown on the figure. Ages 1year old : n=5/group); age 2.5 years old: n=16/group; age 5 years old: n=13/group.

Figure S5: The microbial pathways in CD progressors' gut microbiota are not significantly different between IgA+ and IgA- bacteria

Heat map of predicted metabolic pathways of CD progressors and healthy controls obtained from PICRUSt analysis after IgA sequencing. Each column represents an individual participant and each row represents a predicted microbial functional pathway. Color Code is shown on the figure. Age 1 years old : n=5/group); age 2.5 years old: n=16/group; age 5 years old: n=13/group.
